# A Soluble Epoxide Hydrolase Inhibitor, 1-trifluoromethoxyphenyl-3-(1-propionylpiperidin-4-yl) Urea, Ameliorates Experimental Autoimmune Encephalomyelitis

**DOI:** 10.3390/ijms22094650

**Published:** 2021-04-28

**Authors:** Deepa Jonnalagadda, Debin Wan, Jerold Chun, Bruce D. Hammock, Yasuyuki Kihara

**Affiliations:** 1 Sanford Burnham Prebys Medical Discovery Institute, La Jolla, CA 92037, USA; djonnalagadda@sbpdiscovery.org (D.J.); jchun@SBPdiscovery.org (J.C.); 2 Department of Entomology and Nematology and UC Davis Comprehensive Cancer Center, University of California, Davis, CA 95817, USA; dbwan1984@gmail.com (D.W.); bdhammock@ucdavis.edu (B.D.H.)

**Keywords:** multiple sclerosis, neurology, neuroinflammation, lipidomics, lipid mediator, bioactive lipids, Ephx2, epoxide, drug discovery, neuropharmacology

## Abstract

Polyunsaturated fatty acids (PUFAs) are essential FAs for human health. Cytochrome P450 oxygenates PUFAs to produce anti-inflammatory and pain-resolving epoxy fatty acids (EpFAs) and other oxylipins whose epoxide ring is opened by the soluble epoxide hydrolase (sEH/*Ephx2*), resulting in the formation of toxic and pro-inflammatory vicinal diols (dihydroxy-FAs). Pharmacological inhibition of sEH is a promising strategy for the treatment of pain, inflammation, cardiovascular diseases, and other conditions. We tested the efficacy of a potent, selective sEH inhibitor, 1-trifluoromethoxyphenyl-3-(1-propionylpiperidin-4-yl) urea (TPPU), in an animal model of multiple sclerosis (MS), experimental autoimmune encephalomyelitis (EAE). Prophylactic TPPU treatment significantly ameliorated EAE without affecting circulating white blood cell counts. TPPU accumulated in the spinal cords (SCs), which was correlated with plasma TPPU concentration. Targeted lipidomics in EAE SCs and plasma identified that TPPU blocked production of dihydroxy-FAs efficiently and increased some EpFA species including 12(13)-epoxy-octadecenoic acid (12(13)-EpOME) and 17(18)-epoxy-eicosatrienoic acid (17(18)-EpETE). TPPU did not alter levels of cyclooxygenase (COX-1/2) metabolites, while it increased 12-hydroxyeicosatetraenoic acid (12-HETE) and other 12/15-lipoxygenase metabolites. These analytical results are consistent with sEH inhibitors that reduce neuroinflammation and accelerate anti-inflammatory responses, providing the possibility that sEH inhibitors could be used as a disease modifying therapy, as well as for MS-associated pain relief.

## 1. Introduction

Lipids are essential nutrients and major constituents of the central nervous system (CNS) [1,2]. Fatty acids (FAs) are the building blocks of a variety of lipid species including phospholipids, sphingolipids, and glycolipids [3]. In humans, saturated FAs (palmitic acid, stearic acid) are de novo synthesized by elongation. However, polyunsaturated fatty acids (PUFAs: i.e., linoleic acid, LA (18:2, Δ^9,12^, ω-6); arachidonic acid, AA (20:4, Δ^5,8,11,14^, ω-6); alpha-linolenic acid, ALA (18:3, Δ^9,12,15^, ω-3); docosapentaenoic acid, DPA (22:5, Δ^4,7,10,13,16^, ω-3); docosahexaenoic acid, DHA (22:6, Δ^4,7,10,13,16,19^, ω-3); eicosapentaenoic acid, EPA (20:5, Δ^5,8,11,14,17^, ω-3)) are not produced directly due to the absence of desaturases that introduce a double bond into FAs distal to the *Δ*9 position. Thus, dietary PUFAs are essential for human beings. PUFAs affect membrane flexibility, fluidity, and stiffness [4]. They also serve as precursors for lipid mediators (or bioactive lipids) [2]. For example, AA is converted into prostaglandins (PGs) by cyclooxygenases (COX-1/2) and terminal enzymes, leukotrienes (LTs) by 5-lipoxygenase (5-LO) and LTA_4_ hydrolase or LTC_4_ synthase, and epoxy-eicosatrienoic acids (EpETrE or EET) by cytochrome P450s (CYPs) [2,5]. These enzymes also competitively metabolize ω−3 PUFAs [6]. PGs, LTs, and other lipid mediators play physiological and pathophysiological roles via cognate G protein-coupled receptors (GPCRs) [7]. Because these lipid mediators show pleiotropic functions in a variety of diseases [8,9,10], drugs targeting this signaling have been developed, such as non-steroidal anti-inflammatory drugs (NSAIDs; aspirin, indomethacin, and diclofenac for treatment of pain and inflammation) and cysteinyl LT receptor antagonists (montelukast and pranlukast for asthma and allergic rhinitis) [9,11,12]. PUFA-derived epoxy FAs (EpFAs including EpETrE; LA-derived epoxy-octadecenoic acid, EpOME; ALA-derived epoxy-octadecadienoic acid, EpODE; DHA-derived epoxy-docosapentaenoic acid, EpDPE; and EPA-derived epoxy-eicosatetraenoic acid, EpETE) show anti-inflammatory effects in treating pain, inflammation, cardiovascular diseases, and other conditions [8,9]. Soluble epoxide hydrolase (sEH; mouse gene name, *Ephx2*) opens the epoxide ring in the EpFAs, resulting in less active, sometimes even toxic and pro-inflammatory dihydroxy-FAs (vicinal diols). Inhibitors targeting sEH have been evaluated including a sEH-selective TPPU (1-trifluoromethoxyphenyl-3-(1-propionylpiperidin-4-yl) urea) and a sEH/COX-2-dual inhibitor (PTUPB; 4-(5-phenyl-3-{3-[3-(4-trifluoromethyl-phenyl)-ureido]-propyl}-pyrazol-1-yl)-benzenesulfonamide) to treat pain, cancer, hypertension, brain and heart diseases [9].

Multiple sclerosis (MS) is a neurological disease that is pathologically characterized by the central nervous system (CNS)-specific demyelination and inflammation [13]. Although the cause of MS still remains unclear, both genetic and environmental factors are highly involved in MS pathogenesis [14]. An animal model of MS, experimental autoimmune encephalomyelitis (EAE) [15], is a useful tool to study MS pathology. This model revealed the abnormality of neuro-immune interactions among autoreactive helper T cells secreting IL-17 (T_H_17) or IFN-γ (T_H_1) [16], B cells [17], and the CNS resident immune competent cells (microglia and astrocytes) [18,19]. We previously applied targeted lipidomics and transcriptomics approaches to EAE spinal cords (SCs), which identified the neuroinflammatory functions of PGE_2_ in EAE pathogenesis [20,21]. EAE studies using knockout (KO) mice demonstrated the involvement of cytosolic phospholipase A_2_α (cPLA_2_α) in disease development [22], PGE_2_ receptors (EP_2_ and EP_4_) [23] and the LTB_4_ receptor (BLT_1_) in T_H_17 differentiation [24], and platelet-activating factor (PAF) receptors in macrophage/microglial phagocytic activity [25,26]. One of the significant contributions of the lipid biology field to the MS research is the approval of several sphingosine 1-phosphate (S1P) receptor modulators (fingolimod, siponimod, ozanimod, and ponesimod) by the U.S. Food and Drug Administration (FDA) [7,27]. Based on biochemical and pharmacological studies, S1P receptor modulators down-regulate S1P receptor 1 (S1P_1_) expression on the cell surface as functional antagonists [28], resulting in sequestration of pathogenic lymphocytes from the circulation to secondary lymphoid organs [29]. Moreover, these drugs inhibit astrocyte activation during EAE development [30]. These studies clearly demonstrated that lipid signaling pathways are druggable for MS patients.

In this study, we tested the efficacy of an sEH inhibitor, TPPU (1-trifluoromethoxyphenyl-3-(1-propionylpiperidine-4-yl) urea) in EAE. We also determined the lipid profiles in EAE SCs and plasma of TPPU-treated mice using a quantitative targeted lipidomics approach [31,32,33,34].

## 2. Results

### 2.1. TPPU Protects Mice from EAE

**Figure 1:**
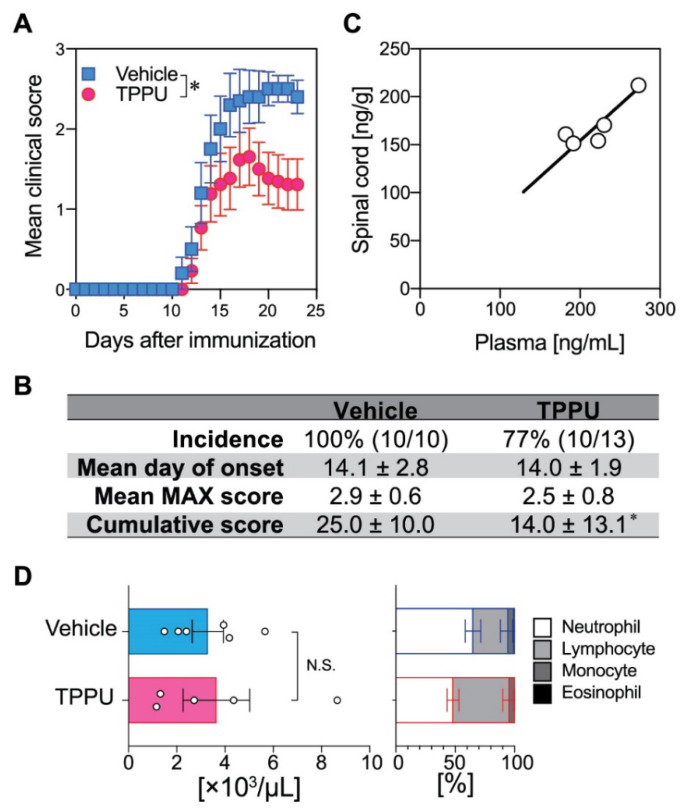
Effect of TPPU on EAE disease course and WBC counts. (**A**) Clinical course of TPPU-treated vs. vehicle-treated EAE mice. (**B**) Clinical parameters of TPPU-treated vs. vehicle-treated EAE mice. Mean MAX score is average of the maximal scores of the mice in each group. (**C**) TPPU concentration in EAE spinal cords and plasma. R^2^ = 0.9708. *P* = 0.0003 was determined by Pearson correlation. (**D**) White blood cell counts and cellular populations in TPPU-treated vs. vehicle-treated EAE mice. *P* values were determined by two-way ANOVA or t-test. N.S., non-significant.

We first tested the efficacy of TPPU, a sEH-selective inhibitor, on EAE development. C57BL/6 female mice were subcutaneously immunized with complete Freund’s adjuvant containing myelin oligodendrocyte glycoprotein peptide (MOG_35–55_) on day 0 and prophylactically treated with TPPU (10 mg/kg, s.i.d.) by oral administration starting from day 0. TPPU treatment significantly ameliorated EAE disease course as compared to controls (treatment, *p* < 0.0001; time, *p* < 0.0001; interaction, *p* = 0.89; by two-way ANOVA) (Figure 1A). Cumulative scores, that were defined as the sum of the clinical scores from days 0 to 23, in the TPPU-treated group were significantly reduced over the vehicle group (Figure 1B). TPPU treatment reduced the incidence and the mean maximal scores (average of the maximal score of the mice in the group), but were not significant (Figure 1B). We detected a considerable concentration of TPPU in both spinal cords (SCs) and plasma, which showed a significant positive correlation (Figure 1C). The white blood cell (WBC) counts and the proportions of WBCs in TPPU-treated EAE mice were equivalent to those in the vehicle-treated EAE mice (Figure 1D). These results suggest that TPPU is effective for treating EAE, and its mechanism of action is different from fingolimod (Gilenya^®^, Novartis), siponimod (Mayzent^®^, Novaritis), ozanimod (Zeposia^®^, Bristol Myers Squibb), and ponesimod (Ponvory^TM^, Johnson & Johnson), which reduce the circulating pathogenic lymphocytes via S1P_1_ down-regulation [7].

**Figure 2:**
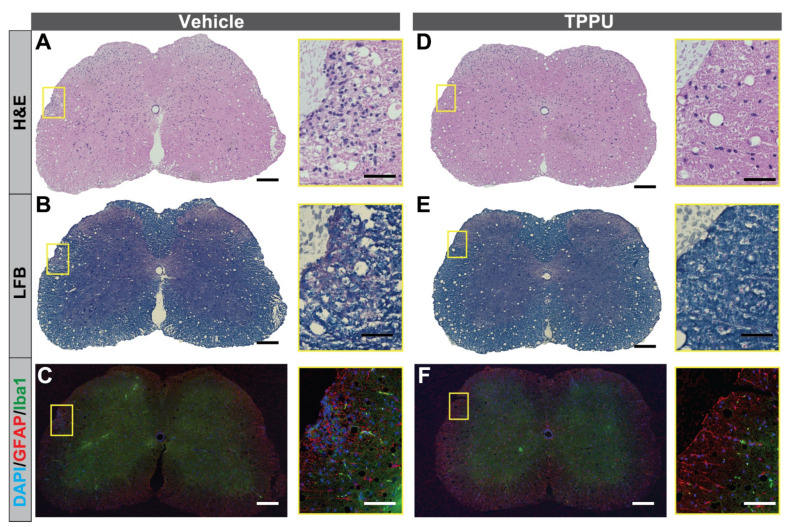
Histological assessment of EAE spinal cords. Representative SC sections of vehicle-treated EAE mice (**A**–**C**) and TPPU-treated mice (**D**–**F**) are shown. (**A**,**D**) H&E staining. (**B**,**E**) LFB-cresyl violet staining. (**C**,**F**) IHC for GFAP and Iba-1. Scale bars = 200 µm. Regions of interest are magnified (Scale bars = 50 µm).

Next, the EAE SCs were stained with hematoxylin and eosin (H&E) and luxol fast blue (LFB)-cresyl violet to assess the degree of inflammation and demyelination (Figure 2). The vehicle-treated group displayed inflammatory cell infiltration into the perivascular regions and parenchyma (Figure 2A), which was associated with myelin pallor (demyelination) and tissue vacuolation (Figure 2B). Importantly, tissue vacuolation was one of the features of EAE and was not observed in the naïve SCs [25]. Immunohistochemstry (IHC) for Iba-1, a microglia/macrophages marker, showed that Iba-1-positive cells accmulated in the EAE lesions and localized in the blood vessels-like structures of the grey matter (Figure 2C). Moreover, astrogliosis was also determined by IHC for glial fibrillary acidic protein (GFAP) (Figure 2C). TPPU treatment showed lesser degree of inflammation, demyelination and astrogliosis (Figure 2D–F) as compared to vehicle controls, while a similar degree of tissue vacuolation was observed between TPPU-treated vs. control groups. The brain RNA-seq database showed specific expression of sEH/*Ephx2* in astrocytes [35], suggesting that TPPU may inhibit astrogliosis as well as inflammation and demyelination.

### 2.2. TPPU Blocked Dihydroxy-FA Production in EAE Plasma and Spinal Cords

We applied targeted lipidomics approaches to analyze lipid profiles in both plasma and SCs of EAE mice that were collected in the chronic phase of EAE. To investigate TPPU effects on lipid metabolism, we first analyzed total lipid levels in the COX, 5-LO, 12/15-LO, and CYP-sEH pathways by calculating the sum of metabolite levels in each pathway. 

**Figure 3:**
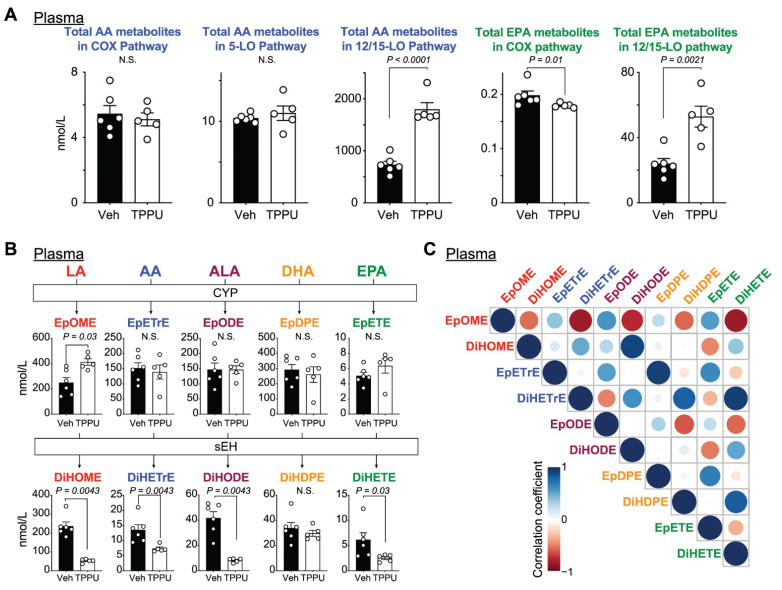
PUFA fluxes in EAE plasma. (**A**) Levels of arachidonic acid (AA) and eicosapentaenoic acid (EPA) metabolites in each pathway. (**B**) Levels of linoleic acid (LA), AA, alpha-linolenic acid (ALA), and docosahexaenoic acid (DHA) metabolites in the cytochrome P450 (CYP)-soluble epoxide hydrolase (sEH) pathway. (**C**) Correlation matrix of EpFAs and dihydroxy-FAs. *P* values were determined by t-test or Mann–Whitney U test. N.S., non-significant.

AA metabolites produced by 12/15-LO were rich in EAE plasma (~1 µmol/L) and were up-regulated by TPPU (~2 µmol/L; Figure 3A). TPPU did not alter COX and 5-LO-mediated AA fluxes, but did significantly reduce COX-mediated EPA metabolites and significantly elevated the 12/15-LO metabolites (Figure 3A). EpFAs were abundantly present (200–300 nmol/L), except for EpETE (~10 nmol/L), in the TPPU-treated and control groups (Figure 3B). As expected from the TPPU inhibitory actions to the sEH, TPPU effectively and significantly blocked the sEH metabolites including dihydroxy-octadecenoic acid (DiHOME), dihydroxy-icosatrienoic acid (DiHETrE), dihydroxy-octadecadienoic acid (DiHODE), and dihydroxy-eicosatetraenoic acid (DiHETE) (Figure 3B). We also found that epoxy-octadecenoic acid (EpOME), a precursor of DiHOME, was significantly elevated in the TPPU-treated group as compared to controls (Figure 3B). Correlation analyses revealed positive relationships within C18-PUFA metabolites and within C20- and C22-PUFA metabolites (Figure 3C). This suggested the association of carbon chain lengths with the substrate preferences in CYPs and sEH activities. All the dihydroxy-FAs showed strong negative correlation with the regioisomeric epoxides of linoleate EpOME (Figure 3C), suggesting a potential anti-inflammatory role for EpOME in EAE or possibly a toxic or inflammatory role for the corresponding diols or DiHOMEs (sometimes termed leukotoxin diols).

**Figure 4:**
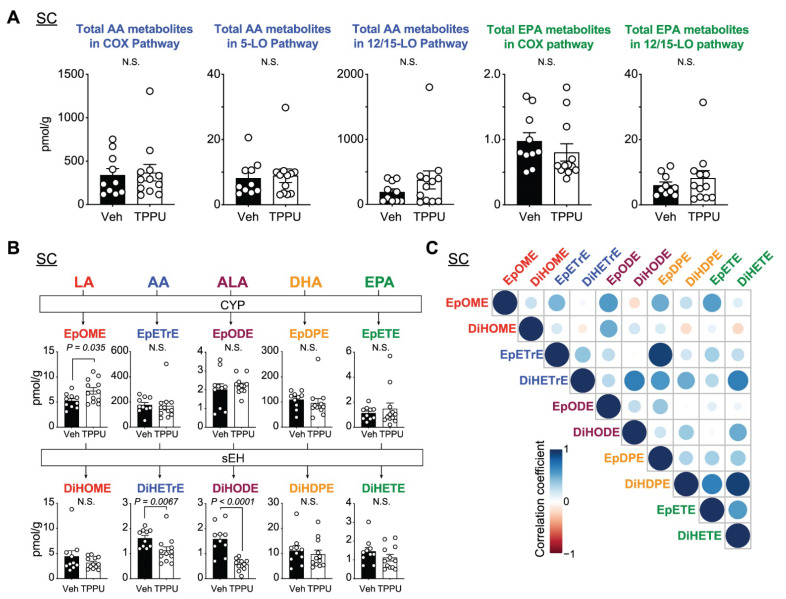
PUFA fluxes in EAE SCs. PUFA fluxes in EAE SCs. (**A**) Levels of AA and EPA metabolites in each pathway. (**B**) Levels of LA, AA, ALA, and EPA metabolites in the CYP-sEH pathway. (**C**) Correlation matrix of EpFAs and dihydroxy-FAs. P values were determined by t-test or Mann–Whitney U test. N.S., non-significant.

In EAE SCs, AA metabolites via the COX-1/2 pathway were abundant (~500 pmol/g). TPPU treatment did not affect fluxes in the COX and 5-LO pathways but showed a similar trend in the 12/15-LO pathway with that of plasma (Figure 4A). Levels of EpETrE and EpDPE (100–200 pmol/g) in SCs were equivalent to those in plasma (100–200 nmol/L), while C18-PUFA metabolites (EpOME and EpODE) were ~50-fold lower than those in plasma (Figure 4B). Similar trends of EpFA and dihydroxy-FA profiles were observed between SCs and plasma, including the inhibition of DiHETrE and DiHODE, as well as an increase of EpOME (Figure 4B) possibly due to TPPU penetrating efficiently into the SCs (Figure 1B). Positive correlations within C20 or C22-PUFA metabolites were found, such as EpETrE vs. EpDPE and DiHDPE vs. DiHETE (Figure 4C).

### 2.3. TPPU Reduced Dihydroxy-FA Production with an Accompanying Increase of EpFAs in EAE Mice

**Figure 5:**
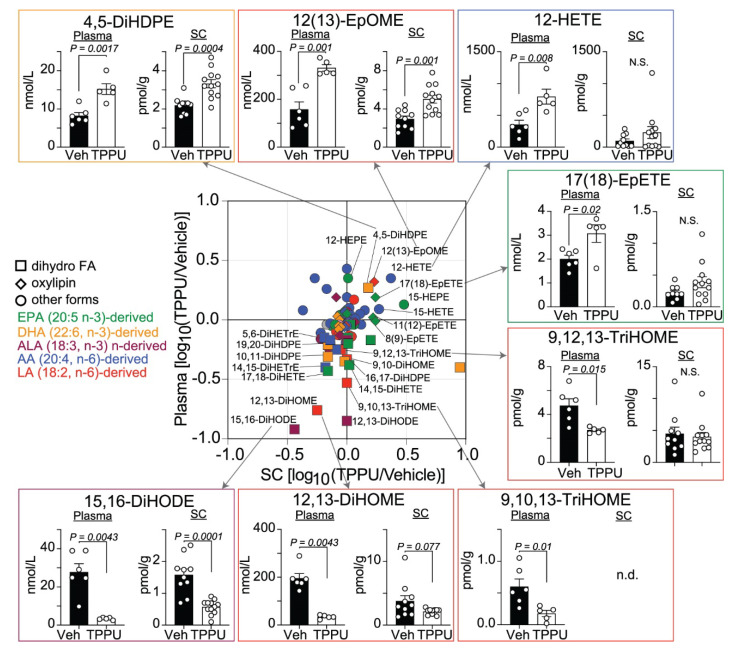
Differential lipid profiles of TPPU-treated vs. vehicle-treated EAE mice. The scatter plot shows the effect of TPPU on lipid levels in EAE SCs (x-axis) and plasma (y-axis). Each symbol represents lipid species coded by color and shape. Representative lipids are displayed as bar graphs. *P* values were determined by t-test or Mann–Whitney U test. N.S., non-significant. n.d., not detected.

Differential lipid levels were computed for TPPU vs. control groups that were represented as a scatter plot (Figure 5). This plot clearly displayed the aggregation of almost all the dihydroxy-FAs (such as 12,13-DiHOME and 15,16-DiHODE) and trihydroxy-FAs (such as 9,10,13-TriHOME and 9,12,13-TriHOME) into quadrant III (log_10_(TPPU/vehicle) < 0 in both plasma and SC) with a few exceptions such as 4,5-DiHDPE (Figure 5). This was accompanied by an up-regulation of some EpFA species including 12(13)-EpOME and 17(18)-EpETE (Figure 5), indicating that TPPU inhibited sEH activity effectively and increased EpFAs consequentially. Furthermore, AA and EPA metabolites in the 12/15-LO pathway (such as 12-HETE) were located in quadrant I of the scatter plot (log_10_(TPPU/vehicle) > 0 in both plasma and SC; Figure 5), suggesting that sEH inhibition caused a potential re-diversion of PUFA to the 12/15-LO pathway, which was also reported in the *Ephx2* deficient mice.

## 3. Discussion and Conclusions

In the present study, we demonstrated the beneficial effect of TPPU in the EAE mice without changing the number of circulating lymphocytes, and also showed that it effectively reduced pro-inflammatory dihydro-FAs in SCs and blood. Currently available disease modifying therapies (DMTs) for MS treatment are mostly immunomodulatory drugs that decrease circulating T and B lymphocytes, and thus prevent pathogenic lymphocytes from penetrating the CNS [36]. DMTs that induce lymphopenia increase serious infection risk, including John Cunningham virus (JCV) infection that causes progressive multifocal leukoencephalopathy (PML) [37]. Although the incidence rates of these infections are reported to be low and similar between DMTs [37], immunomodulatory DMTs appear to be unsafe for use in immunocompromised patients. This study, along with a recent report testing TPPU in EAE [38], provides a novel therapeutic strategy for using TPPU and related sEH inhibitors, which might be effective for all types of MS patients.

sEH inhibitors stabilize most EpFAs studied to date to varying degrees [9]. In general, this is considered beneficial because these epoxides appear to be inflammation resolving agents that reduce ER stress [39]. A recent study showed that TPPU induced neuroinflammatory resolution in a mouse model of Alzheimer’s disease (AD) and increased EpFAs (EpETE and EpDPE) in the brain [31]. However, the findings of the present study were not entirely consistent with these results possibly because of the differences in the MS and AD mechanisms underlying neuroinflammation. We showed that TPPU effectively blocked production of most dihydroxy-FA species, resulting in a compensatory increase of a few EpFA species including 12(13)-EpOME and 17(18)-EpETE (Figure 5). The 12(13)-EpOME (leukotoxin) was believed to be involved in multiple organ failure and adult respiratory distress syndrome until the discovery of the ultimate toxic metabolite, 12,13-DiHOME (leukotoxin diol) [40]. Although sEH induction and subsequent DiHOME production are involved in thermogenesis in brown fat adipose [41], diols of linoleate at high concentrations induce deleterious consequences in vascular and pulmonary permeability [40]. Importantly, the plasma levels of 12,13-DiHOME were associated with severe cases of COVID-19 (coronavirus disease 2019) [42], further supporting the detrimental effects of 12,13-DiHOME in respiratory failure. Since TPPU significantly and robustly reduced the toxic 12,13-DiHOME in EAE plasma and SCs, inhibition of this pathway might be a key mechanism for TPPU’s preventative effects. Moreover, the DiHOMEs should be evaluated as possible biomarkers in EAE, and potentially in MS and related neuroinflammatory diseases.

Anti-inflammatory effects of 17(18)-EpETE have been proposed in several diseases including contact hypersensitivity [43] and non-alcoholic fatty liver disease [44], which may be mediated through one of the three FA GPCRs, GPR40 [43,45], and/or peroxisome proliferator-activated receptor gamma (PPARg) [46]. Although the relative amount of EPA-derived 17(18)-EpETE was small, its increase appeared to be important for neuroinflammatory resolution in EAE. On the other hand, DHA metabolites were mostly down-modulated by TPPU. A unique exception was an increase of 4,5-DiHDPE, whose functions remain elusive, while it most likely shares a similar pro-inflammatory function with other dihdroxy-FAs.

Pharmacological and genetic sEH inhibition appears to alter FA fluxes towards the 12/15-LO pathway. This increased flux may be used for SPM production. Since specialized pro-resolving mediators (SPMs; including lipoxins, hepoxillins, resolvins, protectins) require 12/15-LO activity for their biosynthesis, sEH inhibition may enhance SPM production when substrate PUFAs are sufficiently provided. 12/15-LO deficiency aggravated EAE [47], supporting the pro-resolving and anti-inflammatory effects of 12/15-LO metabolites in EAE and MS. Indeed, resolvin D_1_, which is produced from DHA by the actions of 15-LO and 5-LO, ameliorated EAE [48]. On the other hand, selective sEH inhibition did not affect metabolic pathways mediated through COX-1/2 and 5-LO in either SCs or plasma. Although COX-2 might not be profoundly involved in EAE/MS pathogenesis [49], many eicosanoid species produced downstream of the COX-1/2 and 5-LO pathways show pro-inflammatory action in EAE [20]. Therefore, dual inhibitors for sEH/COX-2 that are currently under development might be beneficial for MS patients and probably more effective in MS-associated pain [10]. TPPU is highly present in the CNS, and its concentration was significantly correlated between SCs and plasma (Figure 1C), supporting a direct action of TPPU in the CNS to suppress neuroinflammation. Taken together, TPPU and other sEH-selective inhibitors appear to be beneficial for the treatment of MS in this murine model and possibly other neurological diseases.

## 4. Materials and Methods

### 4.1. EAE Induction, Treatment and Histology

EAE induction was performed as in previous studies [30,50]. Briefly, 8-week-old C57BL/6 female mice were subcutaneously immunized with 150 µg MOG_35__–55_ peptide emulsified in complete Freund’s adjuvant (BD Biosciences, San Jose, CA, USA cat #463910) containing 4 mg/mL *Mycobacterium tuberculosis* H37Ra (BD Biosciences, San Jose, CA, USA, cat #231141) on day 0. Mice intraperitoneally received 250 ng pertussis toxin (List Biological Labs, Campbell, CA, USA, cat #180) on day 0 and day 2. Clinical scores were recorded daily. TPPU was synthesized as previously described [51] and dissolved in Kollisolv^®^ PEG E 300 (Millipore Sigma, St. Louis, MO, USA, cat #91462). Mice were treated with TPPU (10 mg/kg, p.o., q.d.) from days 0 to 28. SCs were collected and frozen in liquid nitrogen for lipidomics analyses and stored at −80 °C. Blood was collected into K2EDTA-coated tubes (BD Biosciences, San Jose, CA, USA, cat # 365974) by cardiac puncture under deep anesthesia followed by hematological analyses (Allied Analytic, Tampa, FL, USA, Abaxis Vetscan HM2). Plasma was collected and stored at −80 °C for lipidomics analyses.

Spinal cord samples were incubated overnight in 10% neutral buffered formalin (PROTOCOL^TM^, Thermo Fisher Scientific, Waltham, MA, USA) at room temperature. As previously described [52], samples were embedded in warm 2% agar (BD Biosciences, San Jose, CA, USA) and 2.5% gelatin mixture dissolved in water, and were allowed to solidify on crushed ice. The solid block was stored in 70% EtOH, washed with 95% absolute EtOH, 100% absolute EtOH:Xylene (1:1), Xylene, molten warm paraffin (Tissue-Tek, SAKURA, Japan, cat #4005), and embedded into paraffin blocks using manual paraffin embedder (Tissue-Tek, SAKURA, Japan,). Sections (10 μm) were cut using a microtome (Leica RM2155) and used for hematoxylin and eosin, luxol fast blue staining and immunostaining. Antigen retrieval was performed with Diva Decloaker (Biocare Medical, Pacheco, CA, USA) followed by incubation with primary antibodies, rabbit anti-Iba1 (1:500 dilution, Wako, Japan, cat #019-19741) and chicken anti-GFAP (1:1000, Neuromics, Edina, MN, USA, cat #CH22102) in antibody diluent (Dako, Santa Clara, CA, USA, cat #S3022) followed by incubation with secondary antibodies conjugated with Alexa Fluor 488 or Alexa Fluor 568 (1:2000 dilution, ThermoFisher, Waltham, MA, USA, cat #A11008 or cat #A11041, respectively) and counterstained with DAPI (1:10,000 dilution, Sigma, St. Louis, MO, USA, cat #D8417). Sections were visualized and images were acquired on Keyence BZ-X800 microscope.

### 4.2. Lipidomics

TPPU measurements and lipidomics analyses were conducted using a 4000 QTRAP LC-MS/MS instrument (Applied Biosystems Instrument Corporation, Norwalk, CT, USA) as previously described [31,32]. For TPPU measurements in plasma, samples were diluted 10-fold with EDTA solution (0.1% EDTA, 0.1% acetic acid) and 1-volume of internal standard and TPPU-d3 (1 μg/mL in methanol) were added, followed by liquid–liquid extraction by ethyl acetate twice [33]. The extracted samples were dried using a speed vacuum concentrator. The resultant pellet was reconstituted with 100 nM 12-[[(cyclohexylamino)carbonyl]amino]-dodecanoic acid (CUDA) in methanol and subjected to LC-MS/MS analyses.

Spinal cords were homogenized in ice-cold methanol containing 0.1% butylated hydroxytoluene and 0.1% acetic acid. Internal standard mixtures were spiked into the homogenates and stored at −80 °C for 20 h. Samples were processed by solid-phase extraction (OasisHLB Cartridge, Waters, Milford, MA, USA), reconstituted in 200 nM CUDA in methanol, and analyzed by modified LC-MS/MS [34].

### 4.3. Statistics

As appropriate, data were analyzed statistically using Prism software (GraphPad, San Diego, CA, USA) including Student’s t test, Mann–Whitney U test, Fisher’s exact test, Tukey’s multiple comparison test, or two-way repeated measures ANOVA. A *p*-value of <0.05 was considered to be statistically significant.

## Data Availability

The data presented in this study are available on request from the corresponding author.
